# Sudden Cardiac Arrest on the Treadmill: Anomalous Left Main Mimicking Spontaneous Coronary Artery Dissection

**DOI:** 10.1016/j.jaccas.2025.105334

**Published:** 2025-10-08

**Authors:** Thomas Diamond, Cesar Joel Benites-Moya, Abhinav Karan, Khalid Shakfeh, Anvit Reddy, Fabiana Rollini, Ali Zgheib

**Affiliations:** Division of Cardiology, Department of Medicine, University of Florida Health, Jacksonville, Florida, USA

**Keywords:** anomalous aortic origin of the left coronary artery, coronary anomaly, coronary computed tomography angiography, intramural course, sudden cardiac arrest

## Abstract

**Background:**

A 28-year-old healthy woman collapsed while running on a treadmill, with no known past medical history or conditions.

**Case Summary:**

Initial angiography suggested left main spontaneous coronary artery dissection (SCAD), but coronary computed tomography angiography (CCTA) revealed an anomalous left main coronary artery arising from the right sinus with an intramural interarterial course. Surgical correction was performed.

**Discussion:**

This case demonstrates the diagnostic challenges in a young patient presenting with sudden cardiac arrest during exertion, initially presumed to be SCAD; further evaluation with CCTA revealed no evidence of SCAD. The imaging uncovered an anomalous origin of the left main coronary artery from the right coronary sinus with an interarterial and intramural course, which is a high-risk anomaly associated with sudden cardiac death at a young age.

**Take-Home Messages:**

Anomalous courses of the left main coronary artery are rare and associated with sudden cardiac death, particularly in young individuals. CCTA is the gold standard for diagnosis, with surgical intervention being the definitive treatment.

## Graphical Abstract

**Figure:**
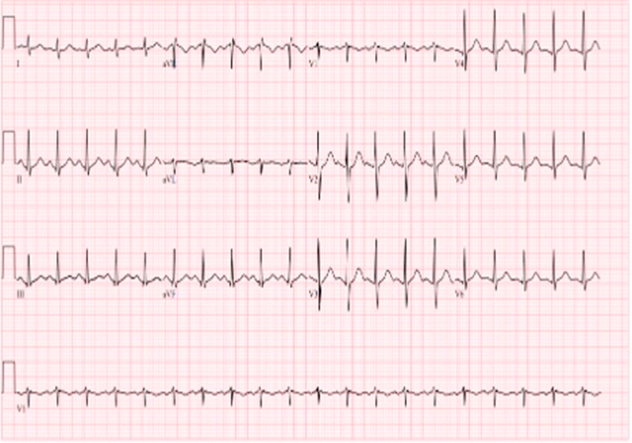

## History of Presentation

**Figure 1:**
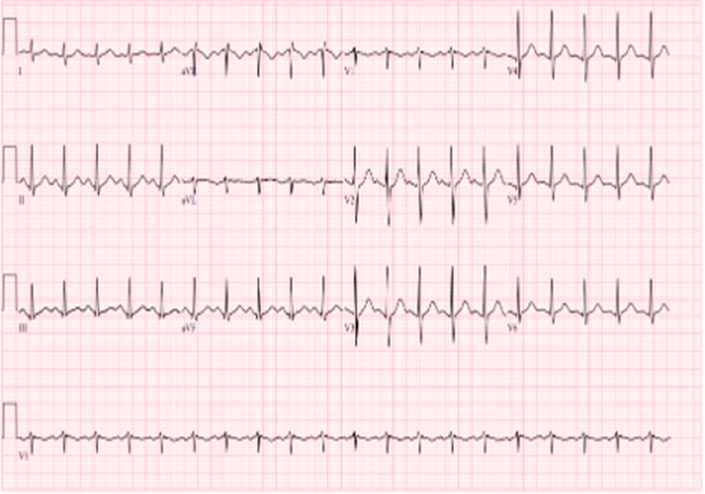
Electrocardiography Demonstrated Sinus Tachycardia Without Ischemic Changes

A 28-year-old woman with no known past medical history collapsed while running on a treadmill. Bystanders initiated cardiopulmonary resuscitation immediately. The initial rhythm was ventricular fibrillation, successfully defibrillated by emergency medical services, followed by a brief episode of pulseless electrical activity. She received 1 dose of epinephrine, with subsequent return of spontaneous circulation. The patient was intubated and sedated in the field. On arrival to the emergency department, electrocardiography (Figure 1) demonstrated sinus tachycardia without ischemic changes. Initial laboratory work-up showed mild anemia, normal renal function, normal electrolytes, and a negative pregnancy test.

**Take-Home Messages**

• Anomalous courses of the LMCA are rare and associated with sudden cardiac death, particularly in young individuals.
• CCTA is the gold standard for diagnosis, with surgical intervention being the definitive treatment.

## Past Medical History

The patient was previously healthy with no known medical conditions. Per her sister, the patient had significant sleep deprivation in the days preceding the event due to examinations and reported frequent consumption of coffee and energy drinks. There was no family history of structural heart disease, arrhythmia, or sudden cardiac death.

## Differential Diagnosis

In a young, previously healthy woman with ventricular fibrillation arrest after exertion, the differential included primary electrical disorders such as catecholaminergic polymorphic ventricular tachycardia, Brugada syndrome, long QT syndrome, and idiopathic ventricular fibrillation. Structural etiologies included hypertrophic cardiomyopathy, arrhythmogenic right ventricular cardiomyopathy, severe myocardial bridging, malignant anomalous coronary arteries, or spontaneous coronary artery dissection (SCAD). Acquired etiologies included stimulant-induced arrhythmia from excessive caffeine and energy drink intake and electrolyte abnormalities from excessive exercise.

## Investigations

**Figure 2:**
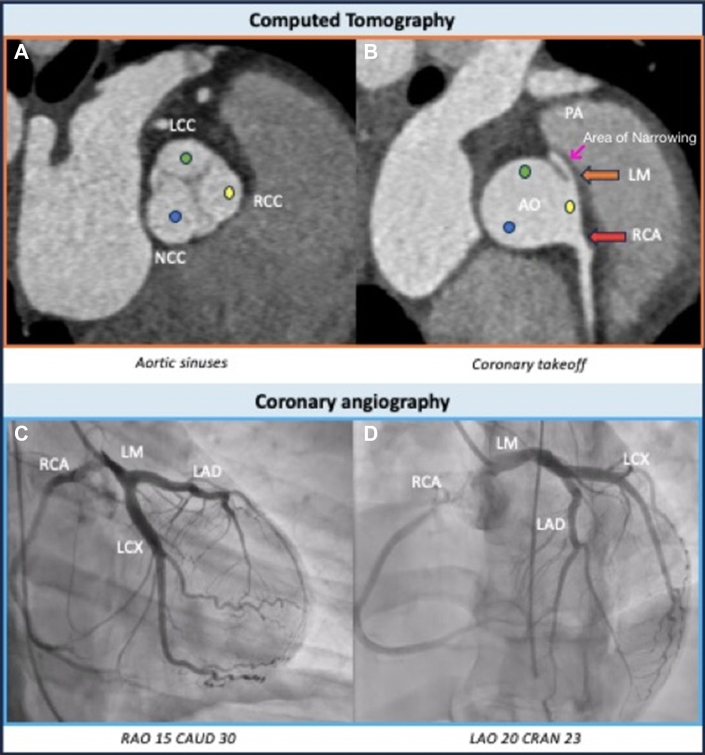
Coronary Angiography Demonstrated Proximal Mid-Left Main Coronary Artery Narrowing, Concerning for Spontaneous Coronary Artery Dissection Ao = aorta; CAUD = caudal view; CRAN = cranial view; LAD = left anterior descending artery; LAO = left anterior oblique; LCC = left coronary cusp; LCx = left circumflex artery; LM = left main; NCC = non coronary cusp; PA = pulmonary artery; RAO = right anterior oblique; RCA = right coronary artery; RCC = right coronary cusp.

Initial high-sensitivity troponin T was elevated at 679 ng/L and down trended on serial measurements. Left heart catheterization revealed a left ventricular ejection fraction of 30%, with anterolateral wall akinesis noted on ventriculography performed in the right anterior oblique 30° fluoroscopic 2-chamber view (Video 1). Coronary angiography demonstrated proximal mid-left main coronary artery (LMCA) narrowing with a possible flap, concerning for SCAD (Figure 2, Video 2). Transthoracic echocardiography showed a left ventricular ejection fraction of 40% with severe hypokinesis of the mid-distal anterior, anterolateral, and inferolateral walls. Coronary computed tomography angiography (CCTA) was performed to evaluate for SCAD and assess for the progression of the left main intramural hematoma. Unexpectedly, CCTA showed no evidence of SCAD but revealed an anomalous origin of the LMCA from the right coronary sinus with an intramural and interarterial course (Figure 2).

## Management

After stabilization, the patient was successfully extubated and initiated on a beta-blocker and aspirin. Given the initial concern for SCAD, P2Y_12_ inhibitors, anticoagulation, and statin therapy were withheld to reduce the theoretical risk of intramural hematoma propagation. After CCTA confirmed the absence of SCAD and revealed a malignant intramural course of the LMCA, a multidisciplinary heart team discussion recommended surgical correction. On hospital day 10, the patient underwent cardiac surgery, which included patch augmentation of the LMCA and anatomic repair of the coronary anomaly. Intraoperatively, the LMCA ostium was found at the commissure between the left and right coronary cusps, and the extramural course was not amenable to unroofing. A vertical incision was made in the left coronary sinus, and an arteriotomy was extended through the LMCA to the level of the sinus dissection. These incisions were connected at their base to ensure complete intimal-to-intimal contact inferiorly. A greater saphenous vein graft was used to perform patch angioplasty of the LMCA to the left coronary sinus, and intraoperative assessment confirmed vessel patency.

## Outcome and Follow-Up

A limited transthoracic echocardiogram obtained before discharge demonstrated recovery of left ventricular ejection fraction to 55% to 60%, with no wall motion abnormalities. The patient was discharged 3 days postoperatively, with a total hospital stay of 13 days. At outpatient follow-up 3 days after discharge, she remained asymptomatic and in stable condition.

## Discussion

This case demonstrates the diagnostic challenges in a young patient presenting with sudden cardiac arrest during exertion, initially presumed to be SCAD. The working theory was that intense physical activity triggered an LMCA SCAD, resulting in complete coronary occlusion and ventricular fibrillation. Cardiopulmonary resuscitation, defibrillation, and pharmacologic support were thought to have restored perfusion by displacing the flap, as evidenced by regional wall motion abnormalities (anterolateral akinesia) on transthoracic echocardiography and ventriculography, consistent with transient ischemia.

However, further evaluation with CCTA revealed no evidence of SCAD. Instead, the imaging uncovered an anomalous origin of the LMCA from the right coronary sinus with an interarterial and intramural course, which is a high-risk anomaly associated with sudden cardiac death at a young age.^3^ The LMCA demonstrated an acute angle of takeoff, a long intramural segment (approximately 13 mm), and coursing between the aorta and pulmonary artery, susceptible to dynamic external compression, mainly during excessive exercise. This likely caused myocardial ischemia and malignant arrhythmia in our patient.

Although CCTA played a key role in reaching the correct diagnosis in this case, it is important to recognize its limitations when it comes to diagnosing SCAD. Compared with invasive coronary angiography, CCTA has lower sensitivity, mainly because it lacks the spatial resolution needed to detect subtle dissections or intramural hematomas, especially in smaller or motion prone coronary segments. For this reason, coronary angiography ideally combined with intravascular ultrasound or optical coherence tomography remains the gold standard for evaluating suspected SCAD.

Intravascular imaging with intravascular ultrasound or optical coherence tomography is often valuable in suspected SCAD because it can reveal dissection planes, intramural hematomas, or subtle flaps that may not be apparent on angiography. In this case, however, we decided not to perform intracoronary imaging due to the high-risk anatomy involving the left main and the potential risk of worsening a dissection or causing additional vessel trauma, especially in the immediate postarrest setting.

In addition to making the diagnosis, CCTA was important for surgical planning. It outlined the intramural and interarterial course of the LMCA, the angle at which it originated, and its relationship to the surrounding great vessels. This helped the surgical team choose the best approach for the arteriotomy and plan the patch augmentation.

Anomalous coronary arteries are extremely rare, with an estimated incidence of 0.3%.^1^ These variants may be benign or malignant, depending on their course. Life-threatening symptoms such as arrhythmias, syncope, myocardial infarction, and sudden cardiac death are estimated to occur in 20% of patients with these anatomic variants.^2^ In particular, a left coronary artery arising from the right sinus is thought to be more strongly associated with sudden cardiac death.^1^ Although coronary angiography can diagnose these conditions, CCTA has become the gold standard for diagnosis because it allows the anomalous course to be completely evaluated.^1^^,^^2^ According to the current adult congenital heart disease guidelines, surgical intervention for such a course is a class 1 indication.^1^

## Conclusions

This case demonstrates the importance of considering anomalous coronary artery origins in young patients presenting with exertional cardiac arrest, particularly when initial angiographic findings are inconclusive. Multimodality imaging, especially CCTA, is essential for diagnosis and surgical planning. In select cases, early use of CCTA probably as a first-line diagnostic imaging too may expedite identification of high-risk congenital anomalies, avoid complications from an invasive approach, and guide timely, lifesaving intervention.

## Funding Support and Author Disclosures

The authors have reported that they have no relationships relevant to the contents of this paper to disclose.

## Appendix

Video 1 Ventriculography Performed in the Right Anterior Oblique 30° Fluoroscopic 2-Chamber View

Video 2 Coronary Angiography Demonstrated Proximal Mid-Left Main Coronary Artery Narrowing With a Possible Flap, Concerning for Spontaneous Coronary Artery Dissection
